# Digital Pathology Quantification of the Continuum of Cirrhosis Severity in Human Liver Biopsies

**DOI:** 10.1111/liv.70166

**Published:** 2025-06-16

**Authors:** Louis Petitjean, Li Chen, Xiaofei Zhang, Thomas Schiano, Mathieu Petitjean, Arun J. Sanyal, MariaIsabel Fiel

**Affiliations:** ^1^ PharmaNest Inc. Princeton New Jersey USA; ^2^ Department of Pathology NYU Grossman Long Island School of Medicine Long Island New York USA; ^3^ Division of Liver Diseases, Department of Medicine, Recanati/Miller Transplantation Institute Icahn School of Medicine at Mount Sinai New York New York USA; ^4^ Department of Internal Medicine, Division of Gastroenterology, Hepatology, and Nutrition Virginia Commonwealth University Richmond Virginia USA; ^5^ Department of Pathology Icahn School of Medicine at Mount Sinai New York New York USA

**Keywords:** artificial intelligence, cirrhosis, digital biomarker, digital pathology, fibrosis, image analysis

## Abstract

**Background and Aims:**

Liver biopsy is the gold standard for assessing fibrosis in cirrhotic livers, yet cirrhosis is spatially heterogeneous and continuously remodels. This study evaluates a novel phenotypic digital pathology platform for continuous fibrosis severity quantification and sensitivity to sampling variability.

**Approach and Results:**

Five needle biopsies were collected from 20 HCV‐cirrhotic livers during transplantation. Histological staging used the Laennec (4A–4C) and Beijing (progressive, regressive, indeterminate) systems. Collagen proportionate area (CPA) was measured via computerised morphometry. The FibroNest platform analysed high‐resolution, single‐fibre images to extract 336 parameters, generating a continuous fibrosis severity score (Ph‐FCS) and tailored scores for Laennec (Ph‐FCS(L)) and Beijing (Ph‐FCS(B)) systems. A comparative MASLD cohort (*n* = 73, NASH‐CRN stages) was also included. The range of the Ph‐FCS was broader to cover the cirrhosis spectrum (6.44 units) than from F0 to F3 (5.39 units). Ph‐FCS was less affected by biopsy variability (16.7% ± 1.3%) compared to CPA (47.3% ± 4.5%). Ph‐FCS(L) and Ph‐FCS(B) demonstrated moderate concordance with the Laennec and Beijing stages. Their ability to classify patients into Laennec and Beijing stages was limited (0.610 < AUROCS < 0.789). At best, Ph‐FCS(L) and Ph‐FCS(B) distinguished stages 4A from 4C and P from R with AUROCs of 0.747(95% CI: 0.611–0.879) and 0.798 (95% CI: 0.645–0.929).

**Conclusions:**

Phenotypic digital pathology biomarkers provide robust, continuous measures of fibrosis severity and activity. They enhance traditional staging systems by offering improved resolution and reduced sensitivity to biopsy variability, with potential value in cirrhosis sub‐staging and clinical decision‐making.

**Summary**

- The authors analyse the performance of a digital/computational pathology tool in its ability to adequately represent the spectrum of fibrosis and its severity in cirrhotic patients and at the same time its sensitivity to sampling (liver biopsy) variability.

**Abbreviations**
AI: artificial intelligence
AUROC: area under the receiving operator curve
CoV: coefficient of variation
CPA: collagen proportionate area
HBV: hepatitis B virus
HCV: hepatitis C virus
HOTSURFR: Hepatic Outcomes and Survival Fatty Liver Registry
IRB: investigational review board
MASH: metabolic associated steatohepatitis
MRE: MRI elastography
MRI: magnetic resonance imaging
NASH: non‐alcoholic steatohepatitis
NASH‐CRN: NASH‐Clinical research network
Ph‐FCS: phenotypic fibrosis composite score
qFT: quantitative fibrosis trait
QIA: quantitative image analysis
ROI: region of interest
SE: standard error
STD: standard deviation

## 1 Introduction

Liver biopsy remains the gold standard for evaluating the severity of fibrosis in liver diseases. A significant issue with liver biopsies is the degree of variation that exists within any given liver [1, 2]. This sampling error is crucial for the proper interpretation of biopsy results and for the appropriate design and power of studies that rely on these biopsies.

Multiple histological methods exist for evaluating and staging fibrosis severity in liver biopsies, each tailored to specific aetiologies and broad clinical severity phenotypes, such as pre‐cirrhotic, compensated cirrhosis, and decompensated cirrhosis. Some of the most commonly used staging methods for Hepatitis C include the METAVIR scale [3, 4], Ishak system [5], and Batts‐Ludwig and Scheuer scoring systems [6, 7] all of which use detailed scoring criteria to assess inflammation and fibrosis.

In staging cirrhosis in patients with hepatitis C virus (HCV), the Laennec classification system refines the staging of fibrosis by subdividing stage 4 into 4A, 4B, and 4C based on the extent and severity of fibrosis and nodularity. The Beijing classification [8], initially used for cirrhosis in patients with hepatitis B virus (HBV) and more recently also used for HCV cirrhosis, attempts to evaluate not only the current state of the patient, but also the progression and regression. This was done by staging patient samples as P (progressive), I (indeterminate/stable), and R (regression), according to phenotypes that relate to each phase, such as fibrotic density, nodular formation, and evidence of fibrosis regression activity.

Recently, several digital pathology and artificial intelligence computational methods have been developed and evaluated for fibrosis assessment [9]. These methods aim to automate existing histological paradigms (a classifier method) or discover novel digital pathology biomarkers (a continuous score approach) that could offer increased detection thresholds, diagnostic and prognostic performance, and benefit from the robustness of an automatic method. Novel continuous biomarkers have the advantage of resolving the uncharted histological phenotypes of fibrosis (such as the spectrum of cirrhosis) and detecting subtle changes and variations within categorical stages. However, their clinical validation is extremely challenging because of the lack of established standards, thus requiring clinical outcome studies.

A well‐known example of a continuous digital pathology biomarker is the collagen proportionate area (CPA) [10, 11], calculated as the ratio of collagen pixel area to the total tissue area. CPA is extremely sensitive to preanalytical methods (e.g., staining variability). Studies have shown that patients with liver biopsies and greater CPAs are less likely to show fibrosis regression [11].

In a recently published study, ‘A Comparative Study of Cirrhosis Sub‐staging Using the Laennec System, Beijing Classification, and Morphometry’ [12] a cohort of twenty HCV patients undergoing transplantation had their explanted livers biopsied in the five segments, all performed and staged by the same pathologist (MIF). This study evaluated intra‐liver biopsy variability by staging each sample according to the Laennec and the Beijing classification system, and CPA. Only five of the twenty cases showed the same Laennec substage among the five biopsies, and the remaining 15 cases showed two to three different Laennec substages, prompting the pathologists to choose the highest Laennec substage to adjudicate a final stage for the case. Using the Beijing classification, seven of twenty cases showed the same fibrosis pattern among all five biopsies, all having P pattern. The remaining 13 cases demonstrated heterogeneous fibrosis patterns among the different segments. When there was heterogeneity present, pathologists used the most predominant pattern to adjudicate a stage. The average Coefficient of Variability of the CPA was 47.3%+/− 4.5%. These results show that both staging and traditional computerised method are highly sensitive to biopsy sampling variability.

In this study, we retrospectively used the same cohort to (a) evaluate the performance of the FibroNest Ph‐FCS along the spectrum of fibrosis severity of cirrhotic patients with HCV, (b) characterise the sensitivity of the FibroNest Ph‐FCS to the spatial heterogeneity of fibrosis in a cirrhotic liver, and (c) develop novel digital pathology continuous biomarkers based on the Laennec and Beijing classifications and evaluate their performance.

The FibroNest Ph‐FCS is a continuous digital pathology biomarker used to assess the severity of fibrosis in a continuous way. The development, analytical validation, and clinical validation of this biomarker have been reported previously [13, 14, 15]. The Ph‐FCS is an exploratory biomarker used to evaluate the effect of a therapeutic or clinical intervention in patients. Its utility is evidenced in the detection of intra‐F‐stage improvement [16] and the prognostic of long‐term liver‐related events [17].

## 2 Methods

### 2.1 Cirrhotic HCV Study Cohort and Patient Demographics

The study cohort comprised 16 men and 4 women, with an average age of 61.8 ± 1.4 years. Demographic and clinical data of the study cohort are summarised in Table 1. The average total explanted liver weight was 1180.3 ± 83.6 g. At the time of transplantation, the average platelet count was 74 ± 7.3 × 10^9^/L, illustrating the clinical severity and advanced disease stage of the cohort [12]. Twenty patients with HCV scheduled for liver transplantation were enrolled as part of an IRB‐approved protocol. Five core biopsies were extracted from predefined liver segments (segments 8, 6, 4, 2, and 1) immediately after liver explantation under direct visualisation using a 14‐gauge (width 2.1 mm) Jamshidi needle.

**TABLE 1:** Demographic and clinical data of the study cohort.

Case #	Age/Gender	MELD	SVR	Liver Wt (g)	Plt count (10^9^/L)	T. bilirubin (mg/dL)	Albumin (g/dL)	HCC
1	53/M	9	Y	2014	111	0.2	3.9	Y
2	62/M	42	N	1480	61	31	2.8	N
3	56/F	15	N	1118	86	3.1	3.6	Y
4	54/M	13	N	1027	52	2.2	2.8	Y
5	74/F	33	N	656	82	7.3	3.3	Y
6	71/M	32	N	835	92	15.4	4	N
7	61/M	12	Y	1141	73	2.7	4	Y
8	62/M	17	N	1885	97	0.5	3.1	Y
9	62/M	36	N	968	21	7.5	3.9	Y
10	68/M	40	N	1075	28	3.3	4.6	N
11	62/F	11	Y	1600	146	1.7	3.9	Y
12	64/M	17	N	1111	94	2.6	2.4	Y
13	69/M	7	N	1260	112	0.5	4	Y
14	62/F	14	N	612	61	2.2	2.4	N
15	48/M	23	N	890	42	4.3	2.7	Y
16	58/M	30	N	1258	98	0.6	3.3	Y
17	59/M	39	N	1289	31	13.2	3.6	N
18	63/M	18	N	848	77	4.3	2.8	Y
19	66/M	23	N	1160	58	5.5	2.4	Y
20	61/M	12	N	1378	59	1.5	3.1	Y

*Note:* Demographic and clinical data of the study cohort, highlighting the severity of liver disease and advanced stage of transplantation.

### 2.2 Histology and Digital Pathology

Liver biopsy specimens were fixed in 10% buffered formalin to preserve cellular integrity and prevent autolysis. After fixation, the specimens were embedded in paraffin wax, sectioned at 4–5 μm thickness, and mounted onto glass slides for staining. The samples were then stained with Masson's trichrome stain and digitised at 20X magnification using a Panoramic 250 Flash III instrument (3D‐Histech).

### 2.3 Pre‐Cirrhotic Enrichment Cohort

To compare the dynamic range of fibrosis progression in the cirrhosis spectrum with that generally observed in pre‐cirrhotic conditions, we included a cohort of 73 patients with MASLD (15), diagnosed by histologic assessment of liver biopsy according to the NASH‐CRN criteria by a single pathologist (F0 [21], F1 [17], F2 [20], F3 [15]). Patients were enrolled as part of an IRB‐approved protocol. FFPE sections (~4 μm) of the biopsies were stained with Picro Sirius red (PSR, Abcam Kit ab150681) and imaged at 20X magnification in white light using an Aperio AT2 Digital scanner.

The selection of this pre‐cirrhotic cohort had some methodological limitations. First, the histological stains used differed. This issue was addressed by the preprocessing step of the FibroNest method, as explained below. Second, the aetiology of fibrosis differs from that in the cirrhotic cohort. We decided to accept this approximation and discuss it further in the analysis.

### 2.4 Histological Staging of Cirrhotic Liver Biopsies

#### 2.4.1 Cirrhosis Subclassification by Laennec System

The Laennec system is an advanced iteration of the METAVIR scoring framework, specifically addressing the staging of cirrhosis by dividing it into three distinct substages (4A, 4B, and 4C) based on the qualitative assessment of fibrous septa and nodular regeneration within the liver architecture [18]. Stage 4A is characterised by the presence of mostly thin septa, with occasional occurrences of thick septa and a variable nodule size, indicative of a relatively milder cirrhosis form. Progressing to stage 4B, the liver exhibited at least two thick septa [19]. The most severe stage, 4C, was identified by widespread thick septa and a preponderance of small nodules, indicating significant disruption of the liver structure. This approach to cirrhosis sub staging facilitates a nuanced understanding of disease progression, correlating histological findings with multiple clinical parameters and clinical outcomes [20].

#### 2.4.2 Cirrhosis Subclassification by Beijing Classification

The Beijing classification adopts a dynamic perspective of cirrhosis activity, categorising the condition into predominantly progressive (P), indeterminate (I), and predominantly regressive (R) patterns. The P pattern is marked by expansive and loosely aggregated collagen, denoting ongoing disease progression. Pattern I represents a transitional state with mixed features of progression and regression, reflecting an ambiguous disease course. Finally, the R pattern is characterised by compact fibrous septa, symbolising a regressive or healing phase of cirrhosis. This classification emphasises the temporal changes in liver fibrosis and offers insights into the potential for disease modification in response to therapeutic interventions.

#### 2.4.3 Single Pathologist Histological Staging

Cirrhosis stages by either Laennec system or Beijing classification were assessed by a single experienced liver pathologist (MIF) following the criteria outlined in both the Laennec and Beijing classification systems. Because if the Laennec stages were not the same across the five biopsies of a given liver, the pathologist chose the highest Laennec substage to adjudicate a final stage for the case. Similarly, when there was heterogeneity in the staging with the Beijing system, the pathologist used the most predominant pattern to adjudicate a stage.

The biopsies of the patient from the pre‐cirrhotic cohort were assessed by a single pathologist using the NASH‐CRN staging system (F‐stages).

#### 2.4.4 Biopsy Adequacy and Sample Quality Limitations

A liver biopsy is considered adequate for fibrosis staging when it is at least 15–20 mm long, contains at least 10–11 portal tracts, and is minimally fragmented, as sample fragmentation can impair accurate assessment [21]. Unfortunately, highly fibrotic and nodular tissues such as the ones collected in this study are brittle and have a significant tendency to fragment, thus generating an experimental limitation of the adequacy criteria. In this study, approximately two‐thirds of the cirrhotic biopsies were not adequate but presented sufficient features for staging according to the Laennec and Beijing systems by expert pathologists. Ten biopsies (10%) were highly fragmented (15 to 20 fragments). All the biopsies of the pre‐cirrhotic cohort were adequate.

### 2.5 High Resolution, Single‐Fibre Phenotypic Digital Pathology Biomarkers

**FIGURE 1:**
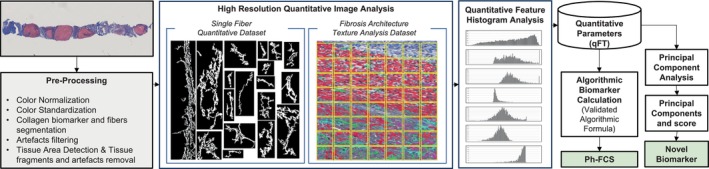
Overview of the FibroNest digital pathology analytical method. The input digital image containing an optical biomarker for collagen (for instance, Masson's Trichrome Stain) is pre‐processed to eliminate tissue and pre‐analytical processing artefacts, non‐contributory features, and to standardise and normalise the colour associated with the collagen biomarker. High‐resolution Quantitative Image Analysis (QIA) is performed to (a) segment collagen objects (or ‘fibres’) that are quantified in multiple morphometric, intensity, and texture features, and (b) quantify fibrosis architecture features from the collagen biomarker image by applying GLCM texture analysis on an optimised distribution of rectangular patterns. Quantitative parameters (qFTs) were extracted from the histogram analysis of each QIA feature distribution. Principal quantitative parameters (principal qFTs) were identified during the biomarker development steps (and accounted for variance between two clinical phenotypes) and were combined in a normalised composite score (the novel biomarker). The Ph‐FCS was established using the principal components and algorithmic formula established in previous development and validation studies.

The digitised images were uploaded to the FibroNest platform, where they underwent the following computational processing steps, as summarised in Figure 1.

#### 2.5.1 Digital Image Pre‐Processing

The aim of the preprocessing step was to correct staining inconsistencies and scanning artefacts. Colour normalisation and standardisation [22, 23] were performed to ensure uniformity across the sample sets. FibroNest automatically identified the biopsy edges to define the region of interest (ROI) for analysis, keeping it 50 μm away from the tissue edge. Anomalies, such as scanning stripes, tissue ruptures, dust, and image compression artefacts, were automatically removed to avoid false signals. The results were visually verified by the operator to correct artefacts, such as small tissue fragments containing more than 90% of fibrosis (as shown in Figure 5, top right image), and to remove non‐contributory fibrotic features, such as fragments of the liver capsule or large, well‐delineated perivascular fibrotic structures. These methods have been used to decrease noise and minimise false signals. Despite the intrinsic nature of cirrhotic livers, where some biopsies were short and highly fragmented, they were still included in automatic digital pathology quantification.

#### 2.5.2 Single Fibre Quantitative Image Analysis

The analytical hypothesis of the FibroNest method is that fibrosis exhibits multiple histological phenotypes according to its aetiology and clinical severity [24]. FibroNest detects and analyzes individual collagen fibres, quantifying their histological traits, including collagen deposition (12 traits), collagen fibre morphometry (13 traits), and fibrosis architecture (seven traits). These traits were statistically described by the histogram of each trait in a biopsy, determining their mean, median, standard deviation, skewness, and kurtosis. Each statistical description represents a continuous parameter known as a ‘quantitative fibrosis trait’ (qFT), which is used to establish a comprehensive quantitative histological phenotypic dataset from an HCV liver biopsy. The full list, nomenclature, and definitions of qFTs have been published elsewhere [24].

#### 2.5.3 Collagen Proportional Area (CPA)

One of the 336 qFTS calculated for the collagen deposition phenotypic layer is the CPA. CPA values in this study will be compared to the CPA values calculated by Zhang et al. [12] from the same cirrhotic cohort et al. using the HALO Collagen Morphometric Module (Indica Labs, Texas USA).

#### 2.5.4 Development of a Continuous Digital Pathology Biomarker for Fibrosis Severity

FibroNest Digital Pathology Fibrosis biomarkers (Ph‐FCS [15, 24]) are a normalised, equi‐weighted, linear combination of ~100 principal qFTs previously identified to account for clinical fibrosis severity. To summarise, principal qFTs were selected from the 336 qFTs if they exhibited a statistically significant change of 20% or more between the early and advanced fibrosis stages. The approach was not based on pathologist annotations but on the definition of clinical severity phenotypes based on conventional clinical evaluations for fibrosis severity (blood panels, Fib‐4, non‐invasive tests).

The development, analytical validation, and clinical validation of this biomarker have been reported previously [13, 14, 15].

#### 2.5.5 Development of a Digital Pathology Biomarker From Laennec and Beijing Cirrhosis Stages

The approach to identify principal qFTs and to construct a normalised composite score and biomarker was modified to model the Laennec and Beijing systems. The Ph‐FCS(L) is assembled with the qFTS, which accounts for the significant variation (principal qFTs, *n* = 135) from the Laennec 4A and 4C biopsy groups (*N* = 23 and *N* = 28, respectively). Similarly, Ph‐FCS(B) was established for the principal qFTs (*n* = 187) identified in the Beijing R and P biopsy groups (*N* = 14 and *N* = 68, respectively). It is expected that the repeatability and reproducibility of these composite scores will be comparable to those established for the Ph‐FCS.

#### 2.5.6 Coefficient of Variation

In this study, the coefficient of variation (CoV [25]) served as a statistical tool to quantify the degree of variability relative to the mean of a dataset. Its purpose is to provide a normalised measure of dispersion, enabling a comparison of the variation in datasets with different units of measure or scales. In this study, it was employed to understand the variations in many of the variables measured. CoV is the ratio of the standard deviation (*σ*) to the mean (*μ*) of the dataset, and is expressed as a percentage:

$$\mathrm{CoV}=\left(\frac{\sigma }{\mu }\right)\times 100$$

A higher CoV indicates a greater level of dispersion around the mean, suggesting higher variability, whereas a lower CoV signifies less dispersion and greater uniformity within the data. Here, we generally consider a CoV below 15% to indicate low variability, between 15% and 30% is considered to indicate moderate variability, and above 30% to indicate high variability [25].

## 3 Results

### 3.1 Liver Biopsy Sampling Variability of the Ph‐FCS and Comparison to CPA%

The CoV of the Ph‐FCS biomarker in relation to intra‐liver spatial biopsy variability (Table 2) was found to be, on average, 16.7% with a standard error (SE) of 1.3%, which is significantly lower than the CoV of Collagen Area Ratio (CAR) (41.4% ± 2.7%) also measured by FibroNest, consistent with previously reported CPA values from the same cohort of 47.3% ± 4.5% [12].

**TABLE 2:** Liver Biopsy (intra‐liver) coefficients of variability from different digital pathology methods.

**Patient ID**	**001**	**002**	**003**	**004**	**005**	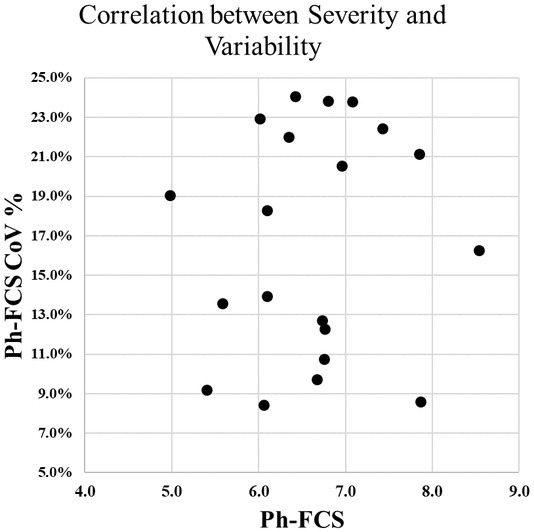
Ph‐FCS(Mean)	5.0	5.4	6.0	7.0	7.8	
Ph‐FCS (CoV%)	19.0%	9.2%	22.9%	20.5%	21.1%	
CPA% (CoV%)	32.4%	27.1%	55.7%	32.4%	44.1%	
**Patient ID**	**006**	**007**	**008**	**009**	**010**	
Ph‐FCS(Mean)	8.5	7.9	7.4	6.8	6.3	
Ph‐FCS (CoV%)	16.3%	8.6%	22.5%	23.8%	22.0%	
CPA% (CoV%)	44.8%	42.8%	64.7%	42.5%	54.8%	
**Patient ID**	**011**	**012**	**013**	**014**	**015**	
Ph‐FCS(Mean)	6.7	6.7	7.1	6.8	6.8	
Ph‐FCS (CoV%)	9.7%	12.7%	23.8%	10.7%	12.3%	
CPA% (CoV%)	28.3%	36.3%	47.7%	21.0%	31.7%	
**Patient ID**	**016**	**017**	**018**	**019**	**020**	
Ph‐FCS(Mean)	6.1	6.1	5.6	6.1	6.4	
Ph‐FCS (CoV%)	18.3%	14.0%	13.6%	8.4%	24.1%	
CPA% (CoV%)	67.6%	45.6%	35.5%	36.8%	36.1%	
	**Mean**	**Median**	**Min–Max**	**Max**	**SD**	
Ph‐FCS(Mean)	6.62	6.70	4.98–8.54	8.54	0.85	
Ph‐FCS (CoV%)	16.7%	17.3%	8.4%–24.1%	24.1%	5.6%	
CPA% (CoV%)	41.4%	39.7%	21.0%–67.6%	67.6%	11.9%	

*Note:* Comparison of the Coefficient of Variation (CoV) between the two digital pathology approaches (Ph‐FSC and CPA%) calculated from the five biopsies of explanted livers of the 20 patients with HCV. For each patient, and collectively, the phenotypic method (multiple qFT embedded in the Ph‐FCS) was significantly less prone to intra‐liver biopsy variability than the single measurement method (CPA%). Here, CPA% is measured by the FibroNest method and is consistent with the CPA% values reported by Zhang et al. (Mean CPA%(CoV%) of 47.3% ± 4.5%). The Coefficient of Variability did not correlate with fibrosis severity.

**FIGURE 2:**
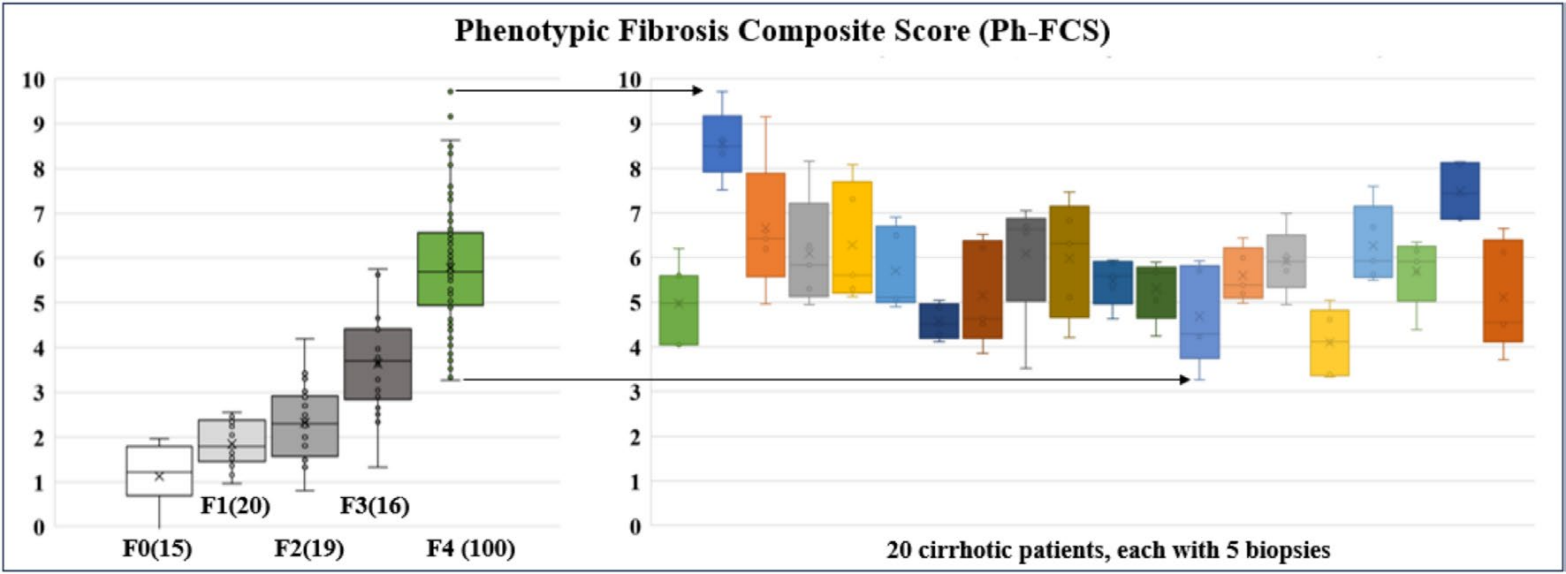
Ph‐FCS correspondence with histological grades of fibrosis severity. Left panel: Correspondence of the FibroNest Digital Pathology biomarker Ph‐FCS with the NASH‐CRN histological grades of fibrosis severity: The spectrum of ‘cirrhosis/F4 severity’ is as extended as the F1 to F3 spectrum. Right panel: Each box plot represents the Ph‐FCS values from the five biopsies performed on the explanted liver. The chart illustrates the liver biopsy sampling variability of Ph‐FCS biomarker severity across liver segments.

The CoV values of Ph‐FCS and CAR for each patient are summarised in Table 2. For each patient, the phenotypic method (multiple qFT embedded in the Ph‐FCS) is significantly more prone to intra‐liver biopsy variability than a single measurement method (CPA). The sampling variability for each patient is shown in Figure 2. These data also established that CoV values were not correlated with the severity of fibrosis (Table 2).

### 3.2 Root Causes of Liver Sampling Variability

**FIGURE 3:**
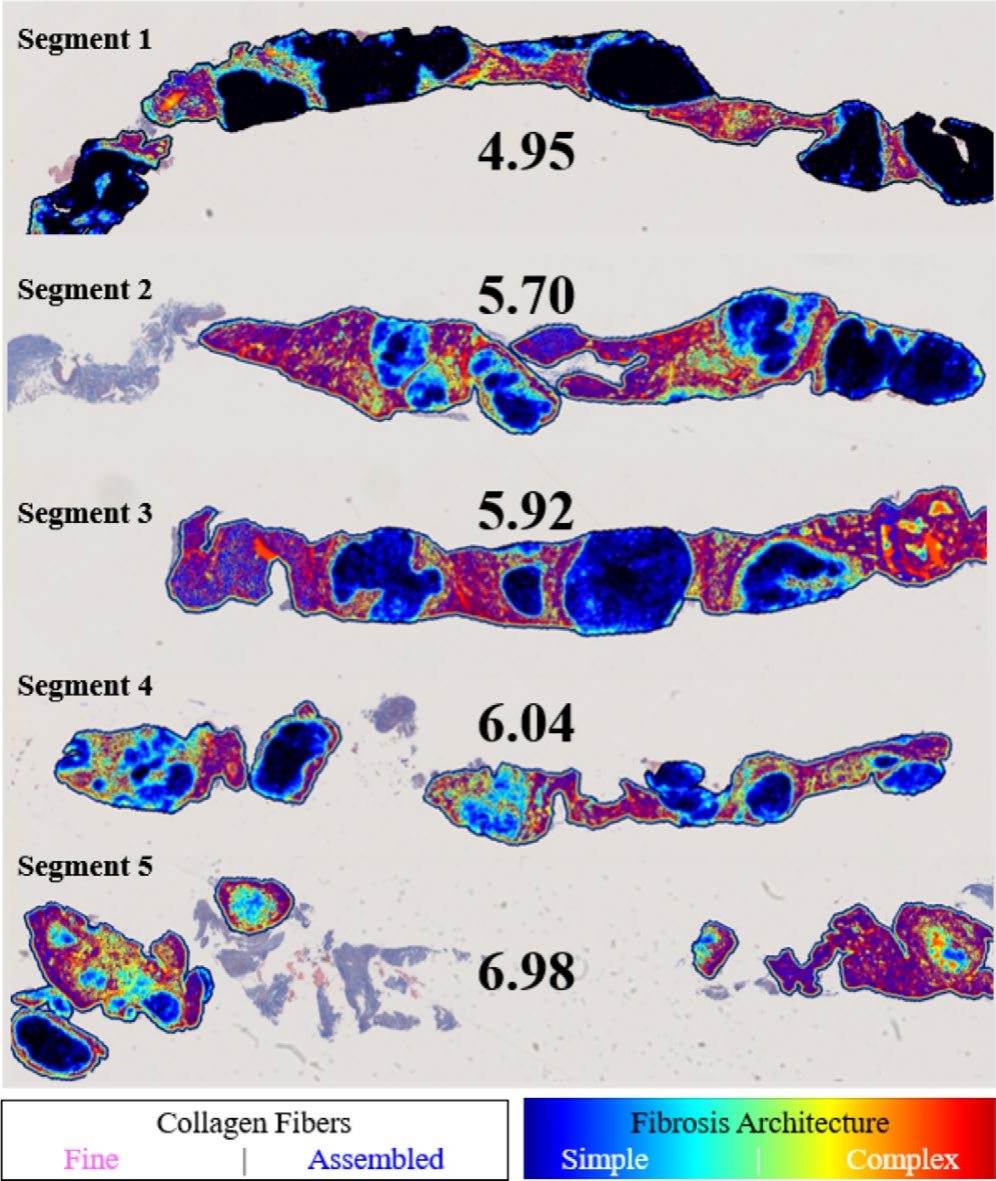
Representative Images of quantitative image analysis. Individual examples (Patient 015) of quantitative image analysis of five biopsies from five different segments of the same explanted liver and corresponding Ph‐FCS scores. Detected fibres were classified and coloured as either assembled or fine fibres based on a branching cutoff for each fibre (assembled > 21 branches, fine < 21 branches). This classification allows for sub‐analyses of the fibre characteristics. An example of an architectural trait (entropy) was used to quantify the degree of complexity of the fibrosis architecture. The composite image illustrates the buildup of fibrosis (light blue‐green) inside the nodules in Segments 2 and 4.

Representative images of five biopsies from different segments of the explanted liver (Patient 015, Figure 2) showed Ph‐FCS values ranging from 4.95 to 6.98, with a Coefficient of Variation (CoV) of 12.3% (Table 2). Figure 3 illustrates that high Ph‐FCS values (6.04 and 6.98) are associated with inadequate, fragmented, and short specimens. These issues often arise because of the brittle nature of highly fibrotic specimens in cirrhotic livers and are not related to regional anatomical features or function. When these inadequate specimens were excluded from the analysis, the CoV decreased from 12.3% to 8.56%.

### 3.3 Continuous Quantification of Fibrosis Severity in the Cirrhosis Spectrum

As shown in Figure 2, the range of Ph‐FCS in the cirrhosis spectrum (6.44, *n* = 100) was higher than that in stages F0 to F3 (5.39, *n* = 73), illustrating both the inadequacy of a single ‘F4’ category to capture cirrhosis and the broad histological diversity associated with cirrhotic liver remodelling.

### 3.4 Ph‐FCS(L) Correspondence With Laennec Severity Stages

**FIGURE 4:**
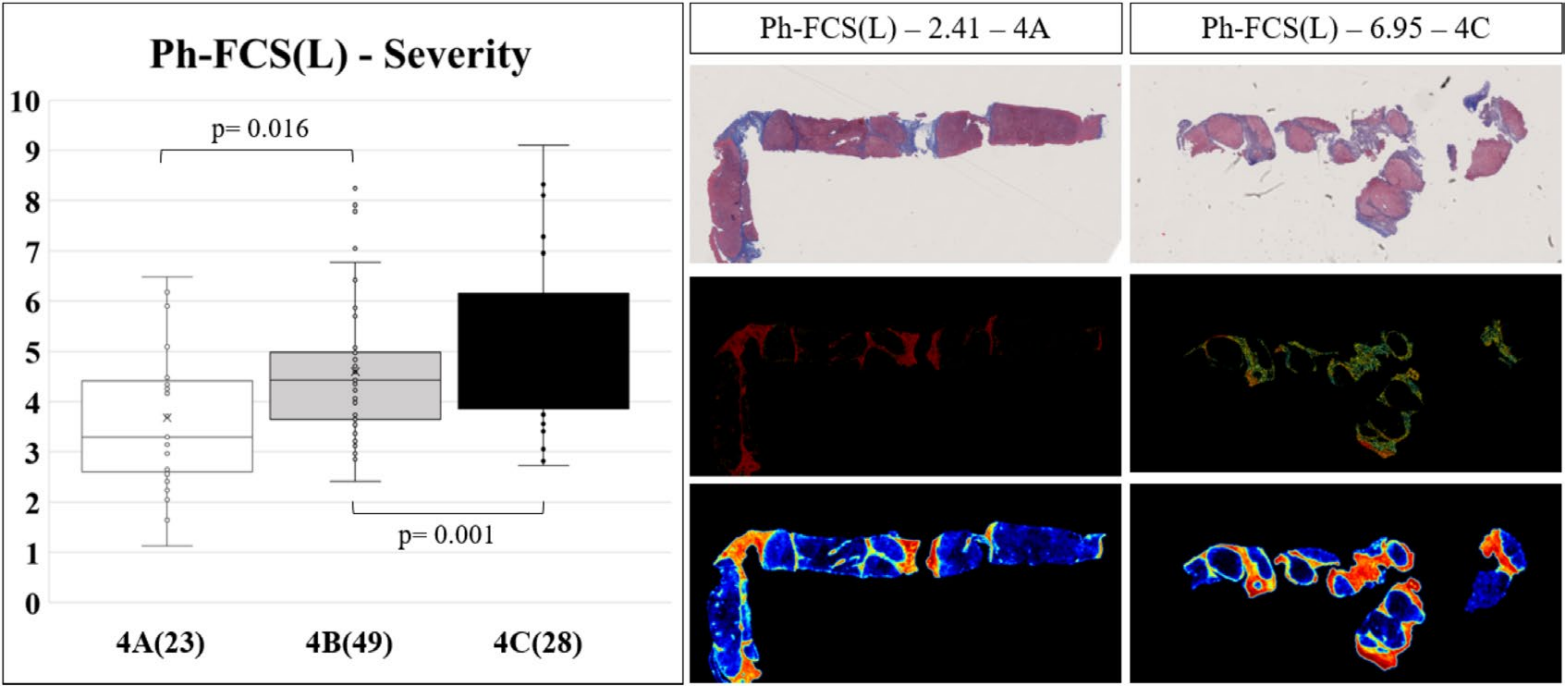
Correspondence of the Ph‐FCS (L) biomarker with Laennec histological grades. The Ph‐FCS(L) for fibrosis severity moderately correlates with Laennec stages, with an AUROC = 0.747 to classify low (4A) from high (4C) fibrosis severity. Representative augmented digital pathology images of Trichrome stains of Laennec liver biopsies with low severity (Ph‐FCS(L) = 2.41, Laennec 4A) and high fibrosis severity (Ph‐FCS(L) = 6.95, Laennec 4C).

The Ph‐FCS(L) exhibits a good concordance with the Laennec system grades (Figure 4, *n* = 100). The Ph‐FCS(L) in the Laennec 4A group (mean 3.68, SE 0.31, *n* = 23) is significantly lower than the Laennec 4B group (4.59, 0.19, *n* = 49, *p* = 0.016) and Laennec 4C group (5.29, 0.34 *n* = 49, *p* = 0.001). The AUROC performance of Ph‐FCS(L) as a diagnostic test of cirrhosis severity was 0.689 (95% CI: 0.541–0.831) to classify 4A from 4B patients, 0.610 (95% CI: 0.475–0.744) to classify 4A from 4B patients, and 0.747 (95% CI: 0.611–0.879) to classify 4A (low) from 4C(high) patients.

### 3.5 Ph‐FCS(B) Correspondence to Beijing Activity Stages

**FIGURE 5:**
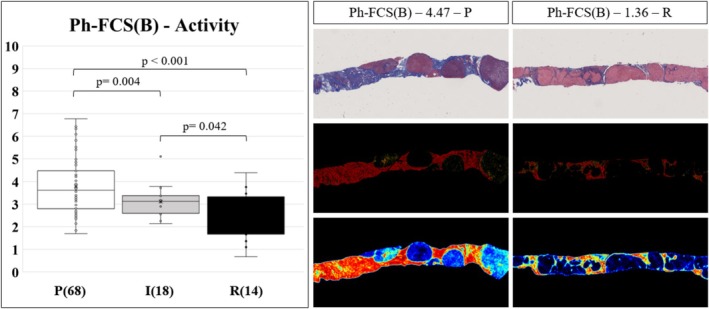
Correspondence of the Ph‐FCS (B) biomarker with Beijing histological grades. The Ph‐FCS (B) for fibrosis activity moderately correlates with Beijing stages, with an AUROC of 0.798, to classify progressive (P) from regressive (R) patients with fibrosis. Representative augmented digital pathology images of trichrome staining of liver biopsies with progressive fibrosis (Ph‐FCS (B) = 4.77, Beijing P) and regressive fibrosis (Ph‐FCS (B) = 1.36, Beijing R).

Similarly, Ph‐FCS(B) exhibited a good concordance with the Beijing system grades (Figure 5, *n* = 100). The Ph‐FCS(B) in the Beijing Progressive (P) group (mean 3.79, SE 0.15, *n* = 68) was significantly higher than that in the Beijing Indeterminate (I) group (3.11, 0.17, *n* = 18, *p* = 0.004) and Beijing Regressive (R) group (2.15, 0.30 *n* = 14, *p* = 0.042). The intermediate (I) stage is best described by 2.5 < Ph‐FCS(B) < 3.5. The AUROC performance of Ph‐FCS(B) as a diagnostic test of fibrosis activity was 0.670 (95% CI: 0.537–0.788) to classify Intermediate from Progressive activity, 0.694 (95% CI: 0.486–0.888), and 0.798 (95% CI: 0.645–0.929) to classify patients with Regressive from Progressive cirrhosis.

### 3.6 Limitations of the Ph‐FCS(L) and Ph‐FCS(B)

The Ph‐FCS(L) and Ph‐FCS(B) scores may suffer from overfitting due to the lack of independent validation, and their performance should be interpreted with caution. Despite this important limitation, the results contribute meaningfully to advancing digital pathology in the context of cirrhosis assessment, even in the absence of a large, well‐characterised cohort with Beijing and Laennec stages.

## 4 Discussion

Despite the advent of non‐invasive methods, such as MRI elastography (MRE) and transient elastography (FibroScan), liver needle biopsy remains the gold standard for assessing fibrosis severity in liver diseases. A key concern with liver biopsies is the intrinsic spatial variability within the liver [1, 2, 26], which introduces sampling errors that can significantly impact the interpretation of biopsy results and the design and power of studies utilising these biopsies.

Histological staging of fibrosis remains crucial, and methods such as the METAVIR and NASH‐CRN [27] scales are widely used, particularly for Hepatitis C (HCV) and MASLD. In cirrhosis, the Laennec and Beijing classification systems have been developed to provide more nuanced staging of severity and capture the dynamic nature of fibrosis progression and regression. These histological systems suffer from inter‐ and intra‐observer variability [28] and lack the granularity to detect subtle changes within fibrosis stages [16].

Digital pathology and computational methods offer promising solutions by potentially automating fibrosis staging or identifying novel histological biomarkers with superior analytical performance [17, 29]. One such historical biomarker, the CPA, has shown sensitivity to fibrosis severity, but is limited by pre‐analytical variability. Computational methods aim to reduce analytical variability and increase detection thresholds; however, their sensitivity to intra‐liver biopsy variability remains a critical question.

Our study aimed to evaluate the FibroNest Ph‐FCS biomarker [15, 30] across the fibrosis severity spectrum, assess its sensitivity to intra‐liver biopsy variability, and develop novel digital pathology biomarkers based on the Laennec and Beijing classifications. The study cohort consisted of patients with MASH and early to severe fibrosis (F0 to F3), and a cohort of 20 cirrhotic patients with HCV who underwent liver transplantation, with five biopsies taken from predefined liver segments. While the aetiology of fibrosis was not the same for all specimen samples, we thought that (i) the benefit of studying this composite cohort outweighed the methodological limitation, and (ii) it only affected a limited section of the results.

We found that the Ph‐FCS range is broader in the cirrhosis spectrum than in the NASH‐CRN stages F0 to F3, underscoring the limitations of a single ‘F4’ category in capturing the full extent of cirrhotic remodelling. This limitation, along with alternative histological frameworks such as the Laennec and Beijing classifications, has been previously highlighted [12, 18, 20, 31]. In this setting, the Ph‐FCS provides a continuous metric that spans the entire fibrosis spectrum—from F0 to advanced cirrhosis—offering potential value for assessing disease resolution and in clinical studies. Nevertheless, sampling variability remains a critical factor when interpreting changes in fibrosis, especially in paired biopsy analyses used to evaluate placebo effects or treatment responses.

To address this question, we also report that the average coefficient of variability for Ph‐FCS due to liver sampling in cirrhotic patients is 16.7% ± 1.3%. This is substantially lower than the variability observed with CPA% reported by Zhang et al. [12] in the same cohort (47.3% ± 4.5%) and the 25%–40% range reported by Bedossa et al. [2, 26] using image analysis methods across all fibrosis stages in specimens over 40 mm in length (up to 100 mm). We attribute this enhanced reproducibility to the phenotypic nature of the FibroNest platform, which integrates ~130 quantitative features across three complementary phenotype layers—collagen deposition, fibre morphometry, and fibrosis architecture—thereby improving the signal‐to‐noise ratio in the Ph‐FCS biomarker.

Consistent with Bedossa's findings [26], the main root causes of sampling variability are related to sampling adequacy, fragmentation, specifically length, area, and anatomical relevance. The exclusion of highly fragmented specimens reduced the CoV of Ph‐FCS by half.

To bridge the gap between traditional categorical staging and emerging continuous digital pathology biomarkers, we developed two phenotypic fibrosis scores: Ph‐FCS(L), reflecting fibrosis severity based on the Laennec system (stages 4A‐B‐C), and Ph‐FCS(B), reflecting fibrosis activity based on the Beijing system (stages P‐I‐R). The ability of these scores to classify patients into Laennec and Beijing stages was moderate, with AUROCs ranging from 0.610 to 0.789. The highest performance was observed in distinguishing stage 4A from 4C using Ph‐FCS(L) (AUROC = 0.747, 95% CI: 0.611–0.879) and P from R using Ph‐FCS(B) (AUROC = 0.798, 95% CI: 0.645–0.929).

These results should be interpreted cautiously. On one hand, the scores were not validated on an independent, well‐curated cohort with established Laennec and Beijing classifications, which would likely yield more conservative AUROC estimates. On the other hand, the sub‐stages were determined by a single pathologist, and potential misclassification or staging uncertainty could have underestimated the true discriminative power of the digital pathology scores. Overall, while promising, the clinical utility of these scores remains limited at this stage. At best, they can be used as an adjunctive biomarker to enhance the Laennec and Beijing system staging method.

To support the interpretation of these novel digital pathology scores, we hypothesise that their analytical performance would be comparable to that of the Ph‐FCS, which has demonstrated repeatability (CoV ~0.3%), reproducibility (CoV ~3.3%), and histological variability related to sectioning and staining (mean ~ 4.5%, ranging from 1.1% to 7.2%) as previously reported [32, 33]. Future studies should aim to validate this hypothesis.

Future studies should also explore the value of combining these quantitative digital pathology phenotypic biomarkers with non‐invasive tests, specifically circulating and imaging biomarkers, into possible composite surrogate biomarkers for the quantification of fibrosis severity and related risks. Such biomarkers could leverage the strengths of each approach and aim to generate diagnostic and prognostic tests with superior performance.

## 5 Conclusion

This study establishes the FibroNest Ph‐FCS digital pathology biomarker as a robust and reproducible tool for quantifying fibrosis severity across the full spectrum of cirrhosis. Compared to conventional CPA measurements (47.3% ± 4.5%), Ph‐FCS demonstrated significantly lower sampling variability (16.7% ± 1.3%), underscoring its reliability in capturing the true heterogeneity of fibrosis in cirrhotic livers. The introduction of continuous phenotypic scores—Ph‐FCS(L) and Ph‐FCS(B), aligned with the Laennec and Beijing systems—further illustrates the power of digital pathology to deliver refined, quantitative assessments of fibrosis severity and activity. While these novel scores show promise as adjunctive tools for evaluating sub‐stages within these classification systems, their standalone diagnostic performance remains limited. Clinical validation continues to be a major hurdle, partly due to the absence of standardised reference cohorts. Nevertheless, the integration of these digital biomarkers with non‐invasive testing strategies holds potential to enhance both diagnostic precision and prognostic insight. Collectively, these findings lay the groundwork for more accurate, automated, and scalable fibrosis staging, with implications for improving clinical decision‐making and therapeutic strategies in liver disease.

## Author Contributions

Concept and design: Louis Petitjean and Li Chen. MariaIsable Fiel and Arun J. Sanyal provided digital pathology images. Analysis and interpretation of data: All authors. Critical study guidance: Mathieu Petitjean and MariaIsabel Fiel. Drafting of the manuscript: Louis Petitjean and Mathieu Petitjean. Critical revision of the manuscript for intellectual content: All authors. Final approval of the version to be published: all authors. Agreement to be accountable for all aspects of the work in ensuring that questions related to the accuracy or integrity of any part of the article are appropriately investigated and resolved: Mathieu Petitjean, MariaIsabel Fiel and Arun J. Sanyal.

## Conflicts of Interest

Louis Petitjean employee of PharmaNest Inc. Mathieu Petitjean, Li Chen are employees and stockholders of PharmaNest Inc. Thomas Schiano, Xiaofei Zhang and MariaIsabel Fiel declare no conflicts of interest. Arun J. Sanyal consultant for Path‐AI, Histoindex, PharmaNest, and Biocellvia. He has stock options in Genfit, Tiziana, Durect, Inversago, and Hemoshear. He has also served as a consultant for Merck, Pfizer, Eli Lilly, Novo Nordisk, Boehringer Ingelhiem, Astra Zeneca, Akero, Intercept, Madrigal, Northsea, Takeda, Regeneron, Genentech, Alnylam, Roche, Glaxo Smith Kline, Novartis, Tern, Fractyl, Inventiva, Gilead, and Target Pharmasolutions. He received royalties from Uptodate and Elsevier. His institution received grants from Intercept, Pfizer, Merck, Bristol Myers Squibb, Eli Lilly, Novo Nordisk, Boehringer Ingelhiem, Astra Zeneca, Novartis, and Madrigal.

## Data Availability

The data that supports the findings of this study are available from the corresponding author upon reasonable request.

## References

[1] V. Ratziu , F. Charlotte , A. Heurtier , et al., “Sampling Variability of Liver Biopsy in Nonalcoholic Fatty Liver Disease,” Gastroenterology 128, no. 7 (2005): 1898–1906. 15940625 10.1053/j.gastro.2005.03.084

[2] P. Bedossa , D. Dargere , and V. Paradis , “Sampling Variability of Liver Fibrosis in Chronic Hepatitis C,” Hepatology 38, no. 6 (2003): ajhep09022. 10.1016/j.hep.2003.09.022 14647056

[3] P. Bedossa and T. Poynard , “An Algorithm for the Grading of Activity in Chronic Hepatitis C,” Hepatology 24, no. 2 (1996): 289–293. 8690394 10.1002/hep.510240201

[4] D. Wendum , K. Lacombe , M. Chevallier , et al., “Histological Scoring of Fibrosis and Activity in HIV–Chronic Hepatitis B Related Liver Disease: Performance of the METAVIR Score Assessed on Virtual Slides,” Journal of Clinical Pathology 62, no. 4 (2009): 361–363. 19126564 10.1136/jcp.2008.062349

[5] K. Ishak , A. Baptista , L. Bianchi , et al., “Histological Grading and Staging of Chronic Hepatitis,” Journal of Hepatology 22, no. 6 (1995): 696–699. 7560864 10.1016/0168-8278(95)80226-6

[6] A. B. Chowdhury and K. J. Mehta , “Liver Biopsy for Assessment of Chronic Liver Diseases: A Synopsis,” Clinical and Experimental Medicine 23, no. 2 (2022): 273–285. 35192111 10.1007/s10238-022-00799-z PMC10224864

[7] K. P. Batts and J. Ludwig , “An Update on Terminology and Reporting,” American Journal of Surgical Pathology 19, no. 12 (1995): 1409–1417. 7503362 10.1097/00000478-199512000-00007

[8] Y. Sun , J. Zhou , L. Wang , et al., “New Classification of Liver Biopsy Assessment for Fibrosis in Chronic Hepatitis B Patients Before and After Treatment,” Hepatology 65, no. 5 (2017): 1438–1450. 28027574 10.1002/hep.29009

[9] V. Ratziu , M. Hompesch , M. Petitjean , et al., “Digital Pathology and Artificial Intelligence in Non‐Alcoholic Steatohepatitis: Current Status and Future Directions,” Journal of Hepatology (2023), 10.1016/j.jhep.2023.10.015. PMC11822446 37879461

[10] M. Israelsen , M. G. Misas , A. Koutsoumourakis , et al., “Collagen Proportionate Area Predicts Long‐Term Mortality in Patients With Alcoholic Hepatitis,” Digestive and Liver Disease 54, no. 5 (2022): 663–668. 34548258 10.1016/j.dld.2021.08.021

[11] E. Buzzetti , A. Hall , M. Ekstedt , et al., “Collagen Proportionate Area Is an Independent Predictor of Long‐Term Outcome in Patients With Non‐Alcoholic Fatty Liver Disease,” Alimentary Pharmacology & Therapeutics 49, no. 9 (2019): 1214–1222. 30882933 10.1111/apt.15219

[12] X. Zhang , T. D. Schiano , E. Doyle , A. D. Branch , S. Florman , and M. I. Fiel , “A Comparative Study of Cirrhosis Sub‐Staging Using the Laennec System, Beijing Classification, and Morphometry,” Modern Pathology 34, no. 12 (2021): 2175–2182. 34381188 10.1038/s41379-021-00881-z

[13] A. Lightstone , L. Chen , and M. Petitjean , “Evaluation of the Performance of AI Digital Pathology Method (FibroNest) on Subsections of Biopsies to Assess Performance Variability due to Region Selection,” Journal of Hepatology 80 (2024): S599.

[14] L. Petitjean , L. Chen , A. Lightstone , and N. Aist , “Novel Digital Pathology Adequacy Score Benchmarks the Performance of Pre‐Analytical Method for Digital Pathology and AI End‐To‐End Tissue Assays,” Journal of Hepatology 80 (2024): S474.

[15] L. Chen , M. Lung , M. Petitjean , C. Behling , and A. Sanyal , “Evaluation of a Novel Histology‐Based Fibrosis Phenotypic Composite Score and Its Correlation With NASH‐CRN Fibrosis Scores in Patients With NASH,” Journal of Hepatology 73 (2020): S421.

[16] V. Ratziu , Y. Yilmaz , D. Lazas , et al., “Aramchol Improves Hepatic Fibrosis in Metabolic Dysfunction–Associated Steatohepatitis: Results of Multimodality Assessment Using Both Conventional and Digital Pathology,” Hepatology (2024): 932–946, 10.1097/HEP.0000000000000980.. 38916482 PMC12186543

[17] L. Chen , L. Petitjean , J. Ampuero , et al., “Novel Artificial Intelligence‐Assisted Digital Pathology Quantitative Image Analysis Predicts the Occurrence of Liver‐Related Clinical Events in the Multicentric, European, Hepatic Outcomes and Survival Fatty Liver Registry (HOTSURFR) Study,” Journal of Hepatology 78 (2023): S651–S652.

[18] S. U. Kim , H. J. Oh , I. R. Wanless , S. Lee , K. H. Han , and Y. N. Park , “The Laennec Staging System for Histological Sub‐Classification of Cirrhosis Is Useful for Stratification of Prognosis in Patients With Liver Cirrhosis,” Journal of Hepatology 57, no. 3 (2012): 556–563. 22617153 10.1016/j.jhep.2012.04.029

[19] D. Jain , P. Sreenivasan , I. Inayat , Y. Deng , M. M. Ciarleglio , and G. Garcia‐Tsao , “Thick Fibrous Septa on Liver Biopsy Specimens Predict the Development of Decompensation in Patients With Compensated Cirrhosis,” American Journal of Clinical Pathology 156 (2021): 802–809. 33940622 10.1093/ajcp/aqab024 PMC8512277

[20] G. Garcia‐Tsao , S. Friedman , J. Iredale , and M. Pinzani , “Now There Are Many (Stages) Where Before There Was One: In Search of a Pathophysiological Classification of Cirrhosis,” Hepatology 51, no. 4 (2010): 1445–1449. 20077563 10.1002/hep.23478 PMC2882065

[21] D. C. Rockey , S. H. Caldwell , Z. D. Goodman , R. C. Nelson , and A. D. Smith , “Liver Biopsy—AASLD Guidline 2009,” Hepatology 49, no. 3 (2009): 1017–1044. 19243014 10.1002/hep.22742

[22] Y. Zheng , Z. Jiang , H. Zhang , F. Xie , J. Shi , and C. Xue , “Adaptive Color Deconvolution for Histological WSI Normalization,” Computer Methods and Programs in Biomedicine 170 (2019): 107–120. 30712599 10.1016/j.cmpb.2019.01.008

[23] F. Pérez‐Bueno , M. Vega , M. A. Sales , et al., “Blind Color Deconvolution, Normalization, and Classification of Histological Images Using General Super Gaussian Priors and Bayesian Inference,” Computer Methods and Programs in Biomedicine 211 (2021): 106453. 34649072 10.1016/j.cmpb.2021.106453

[24] A. Watson , L. Petitjean , M. Petitjean , and M. Pavlides , “Liver Fibrosis Phenotyping and Severity Scoring by Quantitative Image Analysis of Biopsy Slides,” Liver International 44, no. 2 (2023): 399–410. 38010988 10.1111/liv.15768

[25] Wikipedia Commons , “Coefficient of Variation,” Wikipedia, accessed July 18, 2024, https://en.wikipedia.org/wiki/Coefficient_of_variation.

[26] M. C. Rousselet , S. Michalak , F. Dupré , et al., “Sources of Variability in Histological Scoring of Chronic Viral Hepatitis,” Hepatology 41, no. 2 (2005): 257–264. 15660389 10.1002/hep.20535

[27] D. E. Kleiner , E. M. Brunt , M. Van Natta , et al., “Design and Validation of a Histological Scoring System for Nonalcoholic Fatty Liver Disease,” Hepatology 41, no. 6 (2005): 1313–1321. 15915461 10.1002/hep.20701

[28] M. Pavlides , J. Birks , E. Fryer , et al., “Interobserver Variability in Histologic Evaluation of Liver Fibrosis Using Categorical and Quantitative Scores,” American Journal of Clinical Pathology 147, no. 4 (2017): 364–369. 28340131 10.1093/ajcp/aqx011 PMC5848246

[29] V. Ratziu , L. Petitjean , R. Pais , et al., “AI‐Assisted, Quantitative Digital Pathology‐Based Continuous Fibrosis Scores Perform Better Than Conventional Pathology in Documenting Fibrosis Reduction,” Journal of Hepatology 80 (2024): S533.

[30] A. Watson , L. Petitjean , M. Petitjean , and M. Pavlides , Etiology‐Independent Fibrosis Severity Scoring by Quantitative Digital Pathology Image Analysis (International Liver Congress, 2022).

[31] M. Y. Kim , M. Y. Cho , S. K. Baik , et al., “Histological Subclassification of Cirrhosis Using the Laennec Fibrosis Scoring System Correlates With Clinical Stage and Grade of Portal Hypertension,” Journal of Hepatology 55, no. 5 (2011): 1004–1009. 21354227 10.1016/j.jhep.2011.02.012

[32] L. Chen , N. Aist , L. Petitjean , and M. Petitjean , “Evaluation of Performance of AI Digital Pathology on the Reproducibility and Repeatability of Fibrosis Phenotyping in MASH Liver Biopsies,” Journal of Hepatology 80 (2024): S597.

[33] L. Chen , M. Lung , C. Behling , et al., “Evaluation of the Multivendor Performance of a Novel Histology‐Based Fibrosis Phenotypic Composite Score and Its Correlation With NASH‐CRN Fibrosis Scores in Patients With NASH,” Hepatology 74, no. 1S (2022): 953A–954A.

